# Validez y reproducibilidad de un método para estimar la capacidad cardiorrespiratoria en adultos universitarios

**DOI:** 10.7705/biomedica.6404

**Published:** 2022-12-01

**Authors:** Eliana Arcila, Carlos Restrepo, Luis Valbuena, Mario Andrés Quintero, Felipe Marino, Jorge Alberto Osorio, Jaime Gallo-Villegas, Juan Fernando Saldarriaga Franco

**Affiliations:** 1 Grupo de Investigación en Medicina Aplicada a la Actividad Física y al Deporte, Grupo de Investigación GRINMADE, Facultad de Medicina, Universidad de Antioquia, Medellín, Colombia Universidad de Antioquia Universidad de Antioquia Medellín Colombia; 2 Grupo de Investigación en Medicina Deportiva, INDEPORTES Antioquia, Medellín, Colombia INDEPORTES Antioquia Medellín Colombia; 3 Centro Clínico y de Investigación Soluciones Integrales en Riesgo Cardiovascular SICOR, Medellín, Colombia Centro Clínico y de Investigación Soluciones Integrales en Riesgo Cardiovascular SICOR Medellín Colombia; 4 Grupo de Investigación de Epidemiología, Facultad Nacional de Salud Pública, Universidad de Antioquia, Medellín, Colombia Universidad de Antioquia Universidad de Antioquia Medellín Colombia

**Keywords:** capacidad cardiovascular, reproducibilidad de los resultados, pronóstico, consumo de oxígeno, cardiorespiratory fitness, reproducibility of results, prognosis, oxygen consumption

## Abstract

**Introducción.:**

La capacidad física cardiorrespiratoria es un predictor de mortalidad por enfermedad cardiovascular y por todas las causas. Su diagnóstico en diferentes grupos tiene utilidad clínica y en salud pública.

**Objetivo.:**

Evaluar la validez y reproducibilidad de un método sin ejercicio implementado por la NASA (*National Aeronautics and Space Administration*), para estimar el volumen máximo de oxígeno (VO_2_máx) consumido en adultos universitarios.

**Materiales y métodos.:**

Estudio de validación de una prueba que incluyó 94 individuos sanos de ambos sexos (18 a 55 años). La prueba de referencia fue la ergoespirometría. La validez y la reproducibilidad se evaluaron mediante el coeficiente de correlación intraclase (*Intraclass Correlation Coefficient,* CCI) y el método de Bland-Altman*.*

**Resultados.:**

Del total de los individuos incluidos en el estudio, 48,9 % fueron mujeres. La media de edad de los participantes fue de 30,54 ± 9,33 años y, la del VO_2_máx, fue de 41,29 ± 9,54 mlO_2_.kg^-1^.min^-1^. Se encontró una diferencia de medias de VO_2_máx entre la ergoespirometría y el estimado por el método implementado por la NASA de 3,41 ± 5,64 mlO_2_.kg^-1^.min^-1^. La concordancia entre los dos métodos fue buena, con un coeficiente de correlación intraclase de 0,858 (IC_95%_ 0,672-0,926). El porcentaje de error fue del 29,70 %. La reproducibilidad de las dos estimaciones por el método implementado por la NASA fue excelente, con un coeficiente de correlación intraclase de 0,986 (IC_95%_ 0,927-0,995).

**Conclusiones.:**

El método NASA es válido y reproducible para estimar el VO_2_máx en adultos universitarios; además, es seguro y de fácil aplicación. Se recomienda la estimación de la capacidad física cardiorrespiratoria para mejorar la tamización en los programas de riesgo cardiometabólico e implementar intervenciones oportunas.

## Abstract (en)

**Introduction:**

Cardiorespiratory fitness is a predictor of cardiovascular and all-cause mortality. Its assessment in different groups has clinical and public health usefulness.

**Objective:**

To evaluate the validity and reproducibility of a no-exercise method [National Aeronautics and Space Administration (NASA) method] to estimate the maximum oxygen consumption (VO_2_máx) in college adults.

**Materials and methods:**

This study included 94 healthy individuals of both sexes (1855 years). The gold standard was ergospirometry. The validity and reproducibility were evaluated with the intraclass correlation coefficient (ICC) and the Bland-Altman method.

**Results:**

Among the participants, we found a mean age of 30.54 ± 9.33 years and a VO_2_máx of 41.29 ± 9.54 ml O_2_kg^-1^-min^-1^; 48.9 % were women. A mean difference of VO_2_máx between ergospirometry and that estimated by the NASA method of 3.41 ± 5.64 ml O_2_.kg^-1.^ min^-1^ was found. The concordance between the two methods was good, with an ICC of 0.858 (CI_95%_ 0.672-0.926). The percentage of error was 29.70 %. The reproducibility of the two estimates by the NASA method was excellent, with an ICC of 0.986 (CI_95%_ 0.927-0.995).

**Conclusions:**

The NASA method is valid and reproducible to estimate VO_2_máx in college adults. In addition, it is safe and easy to apply. Estimating cardiorespiratory fitness is recommended to improve screening in cardiometabolic risk programs and to implement timely interventions.

La actividad física regular y el ejercicio mejoran la capacidad cardiorrespiratoria ^1^. Además, se asocian con beneficios para la salud. como la disminución en la incidencia de enfermedades crónicas, y el aumento de la calidad y en la expectativa de vida ^2^^-^^4^.

Una escasa capacidad física cardiorrespiratoria es un factor de riesgo independiente de ‘mortalidad cardiovascular y por todas las causas’ ^5^^-^^7^. No obstante, alrededor del 30 % de la población mundial no cumple las recomendaciones de actividad física que permitan obtener beneficios para la salud ^8^^,^^9^. En el mundo, entre el 6 y el 30 % de las muertes por enfermedades crónicas (hipertensión arterial, diabetes mellitus de tipo 2, síndrome metabólico, dislipidemia, enfermedad cardíaca isquémica), pueden atribuirse a una escasa capacidad física cardiorrespiratoria ^10^^,^^11^.

Esta capacidad puede medirse directa o indirectamente ^12^. La determinación directa incluye métodos como: i) calorimetría; ii) sensores de movimiento; iii) acelerómetros, y iv) ergometría con análisis de gases respiratorios. Estos métodos, por su alto costo y complejidad, no son accesibles en algunos contextos clínicos y poblacionales ^6^^,^^7^^,^^12^. La determinación indirecta del máximo volumen de oxígeno (VO_2_máx) consumido puede realizarse por medio de pruebas de laboratorio o de campo. Sin embargo, estas no siempre se pueden usar y, en algunos casos, su validez es cuestionada ^12^.

Se ha promovido el uso de cuestionarios como estrategia alternativa para cuantificar la actividad física que realizan las personas, y hacer vigilancia epidemiológica de las actividades sedentarias ^13^^,^^14^. Entre ellos, el *Global Physical Activity Questionnaire* (GPAQ) de la Organización Mundial de la Salud (OMS) ^13^ y el *International Physical Activity Questionaire* (IPAQ), en sus formas larga y corta ^14^, los cuales han sido usados ampliamente ^15^.

En una revisión sistemática que incluyó 23 estudios de validación de la forma corta del IPAQ, se concluyó que la correlación entre éste y las mediciones objetivas de la actividad física, en la mayoría de los estudios, fue menor que el estándar aceptable; además, con el IPAQ se tiende a sobreestimar la actividad física real hasta en el 84 %. Estos resultados muestran una evidencia débil para justificar el uso de cuestionarios como indicador de la actividad física relativa o absoluta ^15^. También, se ha encontrado variabilidad en la interpretación de las preguntas de los cuestionarios entre individuos de diferentes países, escolaridad y sexo, que ha generado resultados inusualmente altos de actividad física en diferentes grupos poblacionales ^16^^,^^17^.

Con el fin de obtener herramientas sencillas, de bajo costo y confiables para estimar la capacidad física cardiorrespiratoria (un indicador más objetivo de la actividad física), se han desarrollado métodos sin ejercicio; estos incluyen variables de fácil obtención en el consultorio como: i) sexo, ii) edad, iii) índice de masa corporal, iv) frecuencia cardíaca en reposo, y vi) nivel de actividad física autorreportada ^6^^,^^18^^-^^20^. Sin embargo, estos métodos solo han sido validados en algunas poblaciones ^6^^,^^18^^-^^23^. Por lo anterior, el objetivo de este estudio fue evaluar la validez y reproducibilidad de un método sin ejercicio desarrollado por la NASA, para estimar el VO_2_máx en adultos universitarios.

## Materiales y métodos

### Diseño

Se realizó un estudio de validación de una prueba diagnóstica, entre 2016 y 2017, que incluyó 94 personas (estudiantes de pregrado o posgrado, egresados o empleados) pertenecientes a la Universidad de Antioquia en Medellín, Colombia, a quienes se les hizo una evaluación clínica y la medición directa del VO_2_máx, y en quienes se utilizó el método sin ejercicio de la NASA.

### Población

El estudio incluyó adultos sanos, hombres y mujeres, con edades entre los 18 y los 55 años, pertenecientes a la comunidad universitaria. Se excluyeron personas con antecedentes de enfermedad cardiovascular (hipertensión arterial sistémica, malformaciones cardíacas, insuficiencia cardíaca, hipertensión pulmonar, arritmias cardíacas o enfermedad coronaria), consumo de medicamentos (β-bloqueadores, digitálicos o β_2_ adrenérgicos), consumo de sustancias psicoactivas o estimulantes, lesiones o antecedentes de trauma osteomuscular que impidieran la realización de la prueba física, y condiciones neurológicas o mentales que limitaran su participación (anormalidades en la marcha, paresias o plejias, trastornos del sensorio o enfermedad mental descompensada).

### Medición del consumo máximo de oxígeno

La medición del VO_2_máx se realizó de forma directa mediante un espirómetro de circuito abierto Oxycon Delta™ de Jaeger (VIASYS Healthcare GmbH, Hoechberg, Alemania) en banda rodante. Un fisiólogo del ejercicio supervisó la prueba y registró de forma continua el electrocardiograma, la frecuencia cardíaca y la presión arterial. Los participantes hicieron un calentamiento inicial de tres minutos, caminando a una velocidad de 3,5 mph, con un ángulo de inclinación del 1 %. Posteriormente, la velocidad de la banda rodante se incrementó 0,5 mph por cada minuto transcurrido, hasta el agotamiento.

Se consideró una prueba máxima cuando:

- los participantes tenían una percepción subjetiva del esfuerzo mayor de 18, calificada según la escala de Borg en un nivel de 6 a 20;
- se observó una meseta en el consumo de oxígeno a pesar de un incremento en el trabajo o un cociente respiratorio igual o superior a 1,15; y,
- cuando los participantes llegaron a la máxima frecuencia cardíaca prevista.

### Método sin ejercicio para estimar para estimar el consumo máximo de oxígeno

Para la estimación del VO_2_máx se utilizó la ecuación de regresión lineal múltiple, descrita por quienes desarrollaron el método NASA sin ejercicio, que incluye las siguientes variables: i) sexo; ii) edad; iii) índice de masa corporal; iv) frecuencia cardíaca en reposo; y v) el nivel de actividad física autorreportada ^19^ así:

*MET=[sexo x (2,77)-edad x (0,10)-IMC x (0,17)-FCr x(0,03)+CAF x (1,0)]+18,07*

donde MET: tasa metabólica basal en equivalentes; sexo: 1 para hombres y 0 para mujeres; edad: en años; IMC: índice de masa corporal en kg/m^2^;

FCr: frecuencia cardíaca en reposo; CAF: coeficiente de actividad física autorreportada.

En este método de la NASA, se consideran niveles del 1 al 5: el nivel 1 es 0,0 (inactivo, escasa actividad diferente a la de la vida cotidiana); el nivel 2 es 0,32 (participa 5 o más veces por semana, al menos 10 minutos por sesión, en actividades físicas que ocasionan un ligero aumento en la frecuencia cardiaca y respiratoria); el nivel 3 es 1,06 (realiza ejercicio aeróbico, entre 20 y 60 minutos por semana a un ritmo confortable); el nivel 4 es 1,76 (realiza ejercicio aeróbico entre 1 y 3 horas por semana, a un ritmo confortable); y, el nivel 5 es 3,03 (realiza ejercicio aeróbico durante más de 3 horas por semana, a un ritmo confortable). El ejercicio aeróbico incluye caminar, trotar, correr, nadar, montar bicicleta, y practicar deportes vigorosos u otras actividades que requieran un esfuerzo similar.

Se midió la estatura con un tallímetro Seca 2013™ (Seca, Hamburgo, Alemania) y, la masa corporal, con una balanza Omron HBF-510LA™ (Omron Healthcare, Inc., Illinois, USA) con precisión de 0,1 kg.

El porcentaje de grasa corporal fue estimado con la ecuación de Jackson y Pollock ^24^^,^^25^. La frecuencia cardíaca fue medida después de un período de reposo de cinco minutos en posición de decúbito supino, con un pulsómetro Polar^®^ RS800-CX™ (Polar, Kempele, Finlandia). El VO_2_máx fue expresado en ml O_2_.kg^-1^.min^-1^ luego de multiplicar los equivalentes de la tasa metabólica basal (MET) por 3,5. La evaluación para determinar el VO_2_máx estuvo a cargo de un médico especialista en medicina aplicada a la actividad física y al deporte.

### Consideraciones éticas

Se obtuvo el consentimiento informado de cada participante. Se promulgó el respeto, la justicia y la beneficencia a las personas, de acuerdo con las normas científicas, técnicas y administrativas para la investigación en salud del Ministerio de Salud de Colombia en la Resolución 8430 de 1993 ^26^; además, se tuvieron en cuenta los principios de la declaración de Helsinki ^27^. El protocolo de investigación fue aprobado por el Comité de Ética de la Facultad de Medicina, Universidad de Antioquia (Medellín, Colombia).

### Análisis estadístico

Se estimó un tamaño de la muestra de 100 individuos, teniendo en cuenta un coeficiente de correlación intraclase (*Intraclass Correlation Coefficient,* CCI) de 0,7; una amplitud de 0,1; una confianza del 95 % y dos mediciones ^28^.

Inicialmente, se hizo un análisis exploratorio de los datos, con el fin de evaluar la calidad de la información, los valores extremos y los perdidos. Se verificó el supuesto de normalidad mediante la prueba de Kolmogorov-Smirnov. Se describieron las características sociodemográficas, antropométricas y clínicas, según el sexo. En las variables cuantitativas, se presentaron la media y la desviación estándar. Las variables cualitativas se representaron como frecuencias absolutas y relativas.

La concordancia absoluta, variabilidad, sesgo y precisión entre el VO_2_máx medido por ergoespirometría y el VO_2_máx estimado mediante el método sin ejercicio implementado por la NASA, se evaluaron utilizando el coeficiente de correlación de Pearson (r), ICC, coeficiente de variación (CV), raíz del error cuadrático medio (RMSE), diferencia de medias, desviación estándar

(DE) de las diferencias y análisis de Bland-Altman. Asimismo, por medio del método NASA sin ejercicio, se evaluaron la reproducibilidad, variabilidad y concordancia absoluta para dos estimaciones repetidas de VO_2_máx en una submuestra, hechas con una semana de diferencia.

Se construyó un modelo de regresión lineal múltiple a partir de las variables independientes obtenidas del método sin ejercicio utilizado por la NASA, con el fin de determinar la varianza del VO_2_máx obtenido mediante la ergoespirometría. Se calculó el coeficiente de determinación (*R2*) *y* el criterio de información de Akaike como indicador de la bondad de ajuste. Se realizó un análisis de varianza (ANOVA) para establecer si, al menos, un coeficiente β era diferente de 0. De igual manera, se evaluaron la tolerancia y el factor de aumento de la varianza para determinar si existía colinealidad entre las variables independientes. Finalmente, se hizo un análisis residual para verificar el supuesto de normalidad de los datos (prueba de Shapiro-Wilk) y no autocorrelación (estadístico *d* de Durbin-Watson). Se estableció un nivel de significancia estadística α=0,05. Para los análisis, se emplearon los programas IBM SPSS™ Statistics, versión 21.0 (IBM, New York, USA) y Epidate™, versión 4.0. (OPS, Consellería de Sanidade, Xunta de Galicia, España).

## Resultados

En el análisis se incluyeron 94 individuos sanos, con una edad media de 30,54 ± 9,33 años; de estos, el 48,9 % fueron mujeres. El 38,2 % de los participantes se consideraron insuficientemente activos, el 3,2 % reportó consumo de tabaco, mientras que ninguno tenía un patrón excesivo de consumo de licor. La media del índice de masa corporal (IMC) en los hombres fue de 25,79 ± 3,34 kg/m^2^, mientras que, en las mujeres, fue de 24,97 ± 4,65 kg/m^2^. El porcentaje de grasa corporal fue mayor en las mujeres. Según los resultados observados de la frecuencia máxima cardíaca y el cociente respiratorio durante la ergoespirometría, las personas incluidas hicieron un esfuerzo máximo. En toda la muestra, la media del VO_2_máx por ergoespirometría fue de 41,29 ± 9,54 ml/kg/minuto, mientras que el estimado por el método sin ejercicio de la NASA fue 37,88 ± 8,45 ml/kg/minuto. Ell VO_2_máx fue un 22,6 % mayor en los hombres que en las mujeres (cuadro 1). Durante la ergoespirometría, no se presentaron eventos adversos, mayores ni menores.

**Cuadro 1:** Características sociodemográficas, antropométricas y clínicas de los participantes incluidos en el estudio

Variable	Hombres (n = 48)	Mujeres (n = 46)
	Media ± DE	Media ± DE
Edad (años)	30,75 ± 10,46	30,33 ± 8,11
Masa corporal (kg)	77,43 ± 11,06	64,98 ± 13,51
Estatura (m)	1,73 ± 0,07	1,61 ± 0,54
Índice de masa corporal (kg/m^2^)	25,79 ± 3,34	24,97 ± 4,65
Perímetro abdominal (cm)	89,57 ± 10,86	82,33 ± 12,57
Grasa corporal (%)	18,29 ± 7,75	29,16 ± 7,51
Reposo		
Frecuencia cardíaca (latidos/min)	69,29 ± 6,74	71,573 ± 5,6
Presión arterial sistólica (mm Hg)	122,65 ± 7,06	113,65 ± 9,2
Presión arterial diastólica (mm Hg)	73,46 ± 5,93	71,24 ± 5,53
Ergoespirometría		
Frecuencia máxima cardíaca (latidos/min)	186,71 ± 11,52	187,59 ± 9,75
Cociente respiratorio	1,15 ± 0,14	1,15 ± 0,06
Frecuencia cardíaca 1 min de recuperación (latidos/min)	163,69 ± 15,94	164,17 ± 13,20
Frecuencia cardíaca 5 min de recuperación (latidos/min)	121,00 ± 14,42	123,59 ± 15,87
VO_2_máx (ml O2/kg/min)	46,42 ± 9,73	35,94 ± 5,65
Equivalentes de tasa metabólica basal (MET)	13,26 ± 2,78	10,23 ± 1,62
Método NASA		
VO_2_máx (ml O2/kg/min)	42,86 ± 6,32	32,67 ± 7,18
Equivalentes de tasa metabólica basal (MET)	12,25 ± 1,80	9,33 ± 2,05

DE: desviación estándar; VO2máx: consumo máximo de oxígeno

### *Validez de la estimación del VO*_*2*_ *máx mediante el método sin ejercicio en comparación con el obtenido por ergoespirometría*

**Figura 1:**
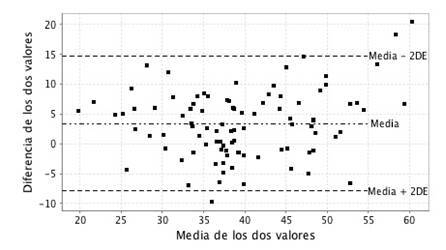
Gráfico de Bland-Altman para evaluar la concordancia del consumo máximo de oxígeno medido por ergoespirometría y el estimado por el método sin ejercicio

La exactitud, precisión, variabilidad y concordancia entre el VO_2_máx medido por ergoespirometría y el estimado por el método NASA sin ejercicio, se muestran en el cuadro 2. Se encontró una diferencia de medias de VO_2_máx entre la ergoespirometría y el método NASA sin ejercicio, de 3,41 ± 5,64 ml O_2_/kg/minuto. La concordancia entre los dos métodos fue buena, con un coeficiente de correlación intraclase de 0,858 (IC_95%_ 0,672-0,926). En la figura 1, se representan los valores de las diferencias frente a la media del VO_2_máx determinada por ambos métodos. Los límites de acuerdo entre las dos técnicas se ilustran mediante las dos líneas punteadas horizontales. Solo tres mediciones del VO_2_máx se encuentran fuera de los límites de acuerdo, sin una tendencia obvia de las diferencias en los valores del VO_2_máx. Finalmente, el porcentaje de error fue del 29,70 %.

**Cuadro 2:** (A) Exactitud, precisión y concordancia de la estimación del consumo máximo de oxígeno mediante el método sin ejercicio en comparación con el consumo máximo de oxígeno medido por ergoespirometría, y (B) reproducibilidad de dos medidas repetidas del consumo máximo de oxígeno mediante el método sin ejercicio

Indicador	Validez	Reproducibilidad
	(A) Ergoespirometría Vs. método sin ejercicio	(B) Método sin ejercicio
Diferencia media (ml O_2_/kg/min)	3,41	-1,28
Desviación estándar de la diferencia (ml O2/kg/min)	5,64	1,50
Límites de acuerdo (ml O_2_/kg/min)	-7,88 a 14,71	-4,28 a 1,72
Porcentaje de error (%)	29,70	7,40
Coeficiente de variación (%)	22,30	20,60
Raíz del error cuadrático medio (L/min)	6,57	1,96
Coeficiente de correlación de Pearson	0,810	0,985
Coeficiente de correlación intraclase	0,858	0,986

### *Análisis de reproducibilidad de la estimación del VO*_*2*_ *máx mediante el método sin ejercicio*

La reproducibilidad, variabilidad y concordancia para dos mediciones repetidas de los valores del VO_2máx_ obtenidos mediante el método sin ejercicio NASA, se muestran en el cuadro 2. La diferencia entre dos mediciones repetidas fue de -1,28 ± 1,50 ml O_2_/kg/minuto. La concordancia entre las dos estimaciones muestra una reproducibilidad excelente con un coeficiente de correlación intraclase de 0,986 (IC_95%_ 0,927-0,995).

**Figura 2:**
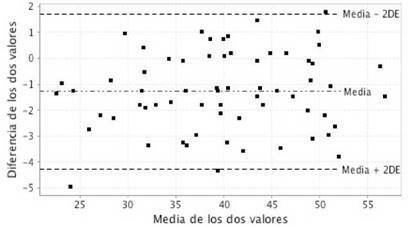
Gráfico de Bland-Altman para evaluar la reproducibilidad del consumo máximo de oxígeno estimado por el método sin ejercicio.

En la figura 2, se representan las diferencias entre las dos estimaciones repetidas frente a la media de las dos estimaciones del VO_2_máx. La línea central muestra la diferencia media y las líneas punteadas representan los límites superior e inferior de los límites de acuerdo. Solo tres estimaciones del VO_2_máx por el método sin ejercicio NASA aparecieron fuera de los límites de acuerdo.

### *Modelo de regresión lineal múltiple: varianza del VO*_*2*_ *máx explicado por las variables independientes del método sin ejercicio*

El modelo de regresión lineal múltiple construido mostró un coeficiente de determinación *R* ^*2*^ de 0,712 y un criterio de información de Akaike de 355,1. El análisis de varianza (ANOVA) mostró que, al menos, un coeficiente β era diferente de 0 (F=40,1; p<0,001). La tolerancia (>0,1) y el factor de aumento de la varianza (<2) mostraron que no había colinealidad entre las variables independientes. El análisis residual mostró cumplimiento de los supuestos de normalidad (prueba de Shapiro-Wilk; z=-0,412; p=0,659) y no autocorrelación (estadístico de Durbin-Watson; *d*=2,27). Los coeficientes β de la actividad física autorreportada, mostraron una relación entre dosis y respuesta desde el nivel 1 hasta el nivel 4 (cuadro 3).

**Cuadro 3:** Modelo de regresión lineal múltiple que muestra la relación entre el consumo de oxígeno medido por ergoespirometría, y la edad, sexo, índice de masa corporal, frecuencia cardíaca en reposo y nivel de actividad física autorreportado

Variable dependiente (consumo máximo de oxígeno por ergoespirometría) (n=94)	β	t	^p^	Intervalo de confianza del 95 %	
(Constante)	17,87	9,56	<0,001	14,15	21,58
Sexo masculino	2,63	7,50	<0,001	1,94	3,34
Edad (años)	-0,09	-4,92	<0,001	-0,13	-0,05
Índice de masa corporal (kg/m^2^)	-0,20	-4,67	<0,001	-0,28	-0,11
Frecuencia cardiaca en reposo (latidos/min)	-0,01	-0,49	0,622	-0,03	0,02
Actividad física autorreportado					
(referencia nivel 1)	0,44	0,76	0,448	-0,71	1,60
Nivel 2	1,00	2,12	0,037	0,06	1,94
Nivel 3	2,16	4,31	<0,001	1,16	3,16
Nivel 4	1,41	2,49	0,015	0,28	2,54
Nivel 5					

## Discusión

Entre los principales hallazgos de este estudio, se encuentran: i) el método sin ejercicio NASA es válido y reproducible para estimar el VO_2_máx en adultos universitarios; ii) el sexo, la edad, el índice de masa corporal, la frecuencia cardíaca en reposo y el nivel de actividad física autorreportada, utilizadas como variables independientes en el método sin ejercicio NASA, explican el 71,2 % de la variabilidad del VO_2_máx; y, iii) los coeficientes β originales del modelo de regresión lineal múltiple del método sin ejercicio NASA, son similares a los encontrados en nuestro estudio.

En el estudio original ^19^, los investigadores desarrollaron y validaron el método sin ejercicio, con el cual se logró estimar la condición física cardiorrespiratoria en individuos caucásicos americanos. En ese estudio, participaron hombres y mujeres entre los 20 y los 70 años que hacían parte de tres cohortes, incluyendo un grupo de empleados de la NASA; de ahí, la denominación del método. El objetivo de ese trabajo fue mejorar, por medio de herramientas simples, el abordaje tradicional para identificar individuos de alto riesgo y asintomáticos, que se beneficiarían de la prevención primaria intensiva.

De la misma forma que en nuestro estudio, los autores de dicho trabajo concluyeron que la condición física cardiorrespiratoria puede estimarse con razonable precisión por medio de un método sin ejercicio, evitando los altos costos y riesgos de la ergoespirometría. Además, encontraron que su modelo predictivo era menos válido en aquellos individuos con un alto nivel de actividad física, lo cual concuerda con nuestros hallazgos; particularmente, en nuestro modelo, el coeficiente β fue menor en la categoría 5 en comparación con la categoría 4 del nivel de actividad física autorreportada. Una posible interpretación de dichos resultados es la sobreestimación que hacen las personas cuando se les indaga por la actividad física que realizan.

En 2013, Sloan, *et al*. ^22^, hicieron una validación cruzada del método sin ejercicio de Jurca y Jackson ^19^ con adultos originarios de Singapur, con el fin de determinar la aplicabilidad de esta herramienta en población asiática, la cual presenta diferencias fisiológicas y genéticas, al compararla con individuos caucásicos americanos. Su objetivo secundario fue hacer una validación cruzada de la ecuación sin la frecuencia cardíaca en reposo, para adaptarla a circunstancias en las cuales no es fácil medirla ^22^. Así como lo detectaron Jurca y Jackson ^19^, y como lo reportado en nuestros resultados, la predicción fue menos válida en sujetos muy activos (que realizan más de 300 minutos por semana de actividad física de moderada intensidad), razón por la cual fueron excluidos del análisis.

Considerando otras aproximaciones para predecir la capacidad física cardiorrespiratoria, en el 2016, O’Donovan, *et al*., publicaron un estudio ^21^. Los autores desarrollaron y validaron ecuaciones para estimar la capacidad física cardiorrespiratoria en 83 hombres blancos europeos y 85 provenientes del sudeste asiático, de edad media, todos residentes en Glasgow y el Reino Unido. Utilizaron variables como i) edad; ii) índice de masa corporal; iii) datos sobre consumo de tabaco; iv) frecuencia cardíaca en reposo registrada mediante electrocardiograma; v) nivel de actividad física medida con acelerómetro; y, vi) origen étnico. Evaluaron de forma objetiva la capacidad física cardiorrespiratoria a partir de la ergoespirometría. Con los resultados obtenidos, indican la utilidad de las variables clínicas y fisiológicas para estimarla. A su vez, subrayan la importancia de incorporar el origen étnico en las ecuaciones con el método sin ejercicio.

En población universitaria también se han utilizado otras metodologías para estimar la capacidad física cardiorrespiratoria ^29^. George, *et al*., realizaron un estudio transversal para validar un método sin ejercicio en el cual se incluyeron 100 estudiantes universitarios (50 % hombres) con edades entre 18 y 29 años ^29^. En este trabajo, utilizaron las siguientes variables independientes: i) actividad física autorreportada; ii) sexo; iii) IMC autorreportado; y, iv) capacidad funcional percibida. Es necesario resaltar que, aunque en esta ecuación no se incluye la frecuencia cardíaca en reposo, los autores reportaron un *R2* similar al observado en el presente estudio (0,720) ^29^.

Una importante aplicación de los métodos sin ejercicio para estimar el VO_2_máx, se da en el contexto de la evaluación y seguimiento de grandes poblaciones, con el fin de evaluar el pronóstico en términos de mortalidad ^30^. Stamatakis, *et al*. ^20^, adelantaron un estudio que incluyó más de 30.000 ingleses y escoses, quienes participaron en ocho encuestas nacionales de salud. La ecuación propuesta por Jurca y Jackson se empleó para estimar la capacidad física cardiorrespiratoria, la cual mostró asociaciones coherentes con la ‘mortalidad cardiovascular y por todas las causas. Asimismo, se halló una buena capacidad de discriminación, y excelentes desempeños en la reclasificación del riesgo de los sujetos. Hallazgos similares son presentados en adultos mayores de 60 años por Song, *et al*. ^31^, quienes reportan que la estimación de la capacidad cardiorrespiratoria (sin una prueba de ejercicio), fue un predictor independiente de mortalidad. Sujetos en el cuartil inferior en cuanto a la capacidad cardiorrespiratoria tuvieron un riesgo 71 % mayor de ‘mortalidad por todas las causas’ (HR=1,71; IC_95%_ 1,30-2,25), en comparación con sujetos en el cuartil superior.

En una investigación que incluyó 43.356 adultos con una mediana de seguimiento de 14,5 años (*The Aerobics Center Longitudinal Study*), Artero, *et al*. ^32^, encontraron que tanto la medición directa de la capacidad cardiorrespiratoria mediante ergoespirometría, como su estimación por un método sin ejercicio (ecuación), se asociaron de forma significativa e inversamente proporcional con el riesgo de mortalidad cardiovascular, eventos coronarios no fatales y mortalidad por todas las causas en hombres; y con la mortalidad por todas las causas y eventos coronarios no fatales, en las mujeres. Mejoras en un MET se relacionaron con reducciones del riesgo de hasta el 20 %.

Zhang, *et al*. ^33^, evaluaron la asociación entre la capacidad física cardiorrespiratoria y la mortalidad, en 12.834 personas con edades entre 20 y 86 años. La estimación se efectuó empleando ecuaciones para hombres y mujeres, con las variables: i) edad; ii) IMC al; iii) circunferencia de cintura; iv) frecuencia cardiaca en reposo; iv) nivel de actividad física autorreportada; y, v) tabaquismo. Se halló que por cada MET de incremento en la capacidad física cardiorrespiratoria, se redujo en el 18 % (rango, 15-21 %) el riesgo de ‘mortalidad por todas las causas’ Los anteriores resultados demuestran la validez del pronóstico, así como la utilidad clínica y en salud pública que puede tener la estimación de dicha capacidad empleando métodos sin ejercicio.

El presente estudio presenta algunas fortalezas, que se pueden agrupar en tres aspectos. En primer lugar, es la validación de un método para estimar la capacidad cardiorrespiratoria sin ejercicio, de fácil obtención (bajo riesgo, costo y tiempo de adquisición), que integra variables sociodemográficas, fisiológicas y de autorreporte del nivel de actividad física; este abordaje supera las limitaciones de los cuestionarios tradicionales sobre la actividad física usados en estudios poblacionales. En segundo lugar, permite incluir personas con un amplio rango de edad, de ambos sexos y con diferentes niveles de capacidad cardiorrespiratoria, sedentarios y activos. En tercer lugar, es un protocolo diseñado por un equipo multidisciplinario, con el liderazgo de especialistas en medicina aplicada a la actividad física y al deporte, y epidemiólogos.

Por otra parte, este trabajo también presenta algunas limitaciones. Con el modelo utilizado, la frecuencia cardíaca en reposo no se asoció con la capacidad física cardiorrespiratoria, lo cual plantea cuestionamientos sobre la veracidad de esta medida, un hallazgo también reportado por otros autores ^21^^,^^22^^,^^34^^,^^35^. Esta situación podría deberse a la variabilidad de la frecuencia cardiaca en reposo en el consultorio médico. Además, otra limitación es la falta de una relación entre dosis y respuesta, entre la actividad física autorreportada y el VO_2_máx, particularmente para el nivel 5, lo cual puede deberse a que las personas tienden a sobreestimar su actividad física.

Finalmente, la capacidad física cardiorrespiratoria es un factor predictor independiente de la mortalidad general y cardiovascular ^5^^-^^7^. Hay intervenciones fundamentadas en programas de ejercicio que incrementan esta capacidad física y disminuyen la morbimortalidad ^1^^-^^4^. Teniendo en cuenta la alta carga por enfermedades crónicas no transmisibles ^36^, es prioritario identificar y utilizar métodos de tamización válidos, seguros y sencillos, que permitan cuantificar la capacidad física cardiorrespiratoria, más allá de los factores de riesgo clásicos ^3^^-^^7^^,^^37^.

En investigaciones futuras, el interés debería centrarse en la evaluación de la validez de las estimaciones de dicha capacidad por métodos sin ejercicio en diferentes grupos poblacionales; de igual manera, deberían incorporar otras variables clínicas y fisiológicas de fácil medición. En una perspectiva avanzada, la capacidad física cardiorrespiratoria podría considerarse como un signo clínico vital para la vigilancia y para la implementación de acciones prioritarias en salud pública ^6^^,^^7^.

El método sin ejercicio NASA es válido y reproducible para estimar el VO_2_máx en adultos universitarios; además, es seguro y de fácil aplicación. Se recomienda la estimación de la capacidad física cardiorrespiratoria para mejorar la tamización en los programas de riesgo cardiometabólico y para implementar intervenciones oportunas.
